# Effects of Replacing Alfalfa Hay With Native Grass Hay in Pelleted Total Mixed Ration on Physicochemical Parameters, Fatty Acid Profile, and Rumen Microbiota in Lamb

**DOI:** 10.3389/fmicb.2022.861025

**Published:** 2022-04-29

**Authors:** Shuai Du, Sihan You, Lin Sun, Xiaolong Wang, Yushan Jia, Yulei Zhou

**Affiliations:** ^1^National Engineering Laboratory of Biological Feed Safety and Pollution Prevention and Control, Key Laboratory of Molecular Nutrition, Animal Nutrition and Feed Science of Zhejiang Province, Ministry of Agriculture, Rural Affairs, and Education, Institute of Feed Science, Zhejiang University, Hangzhou, China; ^2^Key Laboratory of Forage Cultivation, Processing and High Efficient Utilization, Key Laboratory of Grassland Resources, Ministry of Agriculture and Education, College of Grassland, Resources and Environment, Inner Mongolia Agricultural University, Hohhot, China; ^3^Inner Mongolia Academy of Agricultural Science and Animal Husbandry, Hohhot, China; ^4^Branch of Animal Husbandry and Veterinary of Heilongjiang Academy of Agricultural Sciences, Qiqihar, China; ^5^College of Chemistry and Life Sciences, Chifeng University, Chifeng, China

**Keywords:** alfalfa, native grass, meat quality, microbiota, rumen

## Abstract

This study aimed to investigate the effects of replacing alfalfa with native grass on growth performance, meat quality, and rumen microbiota of lambs. Forty-five 6-month-old Ujimqin lambs with live body weight (BW) of 29.50 ± 0.26 kg were used for the experiment, and the lambs were assigned to three diet treatments (three pens per treatment and five lambs per pen) with 30 square meters per pen in semi-open housing based on similar BW. The lambs have received 30% alfalfa (HA, high alfalfa percentage group), 20% alfalfa (MA, moderate alfalfa percentage group), and 10% alfalfa (LA, low alfalfa percentage group) diets, respectively (dry matter basis). The diet treatments had a significant (*P* < 0.05) influence on the dry matter intake of lambs and the dry matter intake increased with the increasing percentages of native grass hay, while the significantly (*P* < 0.05) lower final BW and average daily gain were observed in the MA and LA groups compared with that in the HA group. The diet had a significant (*P* < 0.05) difference in meat pH value, water loss rate, cooked meat rate, moisture, and intramuscular fat, while no significant (*P* > 0.05) difference was found in protein. As native grass hay percentages increased in the diet, the contents of palmitic (C16:0) and palmitoleic (C16:1 *cis*-9) in the HA and MA groups were significantly (*P* < 0.05) lower than that in the LA groups, and compared with the HA group, the contents of elaidic (C18:1 *trans*-9), oleic (C18:1 *cis*-9), and linoleic (C18:2 *cis*-9–*cis*-12) were significantly (*P* < 0.05) increased in the MA and LA groups. The content of α-linolenic (C18:3n3) was significantly (*P* < 0.05) higher in the LA group than that in the HA and MA groups. The principal coordinate analysis profile displayed that the composition of the bacterial community of these groups was distinctly separated from each other. No significant (*P* > 0.05) difference was observed in the dominant rumen bacteria at the phyla and genus levels. In conclusion, the meat quality and fatty acid profile analysis revealed that replacing alfalfa hay with native grass hay is more beneficial for Mongolian lambs, and the meat from LA diet treatment is better than the others. In addition, correlation analysis of the association of rumen microbiome with growth performance, meat quality, and fatty acid profile provides us with a comprehensive understanding of the composition and function of rumen microbiota. These findings could provide knowledge of how the diet affects the animal performance, meat quality of lambs, and microbiota of the rumen, laying a theoretical basis for replacing alfalfa with native grass.

## Introduction

Alfalfa is an excellent forage used for animals, but the availability of this feed ingredient in the global herbivorous animal industry is limited (Hao et al., 2017). The shortage of high-quality roughage and the quantity of imported alfalfa hay cannot meet the needs of the husbandry, and the situation has become increasingly severe, especially in China (Hao et al., 2017; Li et al., 2019, 2020a). Therefore, given China’s current situation, finding suitable forages to replace expensive imported roughage is necessary.

The native grassland is the largest terrestrial ecosystem in China (41% of the total land area), especially in the Inner Mongolia Autonomous Region, which is consistent with meadow steppe, typical steppe, and desert steppe (Jin et al., 2018). But the native grass hay is used as feed for ruminants alone resulting in an imbalance between supply and demand of energy because of the lower nutrient and harsh weather in winter. Previous studies have demonstrated that supplementary with concentrate could improve growth performance and carcass characteristics (Du et al., 2020; Bu et al., 2021). In addition, the pelleted diet-fed lambs could also improve the growth performance and carcass characteristics by enhancing dry matter (DM) and energy intake, which shake off the disadvantages of feeding native grass directly (Du et al., 2020; Zhou et al., 2022). Furthermore, replacing alfalfa hay with dry corn gluten feed and Chinese wild ryegrass can effectively increase the DM intake and promote microbial crude protein (CP) synthesis for ruminants (Hao et al., 2017).

Ruminants provide an abundant source of animal protein products to meet the nutrition demand of the growing population worldwide, because it has a specialized digestive organ, the rumen, which ferments the feed and converts fibrous-rich plant materials and non-human edible plant materials to protein *via* the microbial community (Xue et al., 2020; Chai et al., 2021). This unique microbial ecosystem leads to the development of mutualistic symbiosis between hosts and rumen microbial community composition, which could provide about 70% energy for the ruminant needs (Rosenberg and Zilber-Rosenberg, 2018). Most interestingly, bacteria play important roles in most of the feed biopolymer degradation and fermentation, which indicated that the bacteria are key players to the host than others (Bickhart and Weimer, 2018; Xue et al., 2020). According to a previous report, the rumen community composition is strongly influenced by individual genetics, animal age, feed type, and feeding system (Trabi et al., 2019). Among these factors, the identification of rumen community compositions and functions change are directly linked to the diet (Furman et al., 2020). Previously published studies showed that alfalfa and native grass not only affect the performance and carcass traits but also influence rumen microbiota (Zhou et al., 2022). At present, research on native grass has primarily focused on feeding experiments in lambs, and there are few reports on its use as a replacement for high-quality forages, such as alfalfa hay. The fiber in native grass could supply ruminal microbiota with a readily fermentable source of energy and promote microbial protein synthesis (Li et al., 2020b).

Our hypothesis for this study was that the replacement of alfalfa hay with native grass hay, which is abundant and cheap in China, could provide sufficient physical effective fiber in the diet and improve rumen metabolism and microbial protein production of lambs. Therefore, the objective of this research was to determine the effects on intake, performance, meat quality, and rumen microbiota when a portion of alfalfa hay was replaced with native grass hay in the diets of lambs.

## Materials and Methods

The research protocol used in this study was according to the Institutional Guidelines for Animal Experiments of the College of Chemistry and Life Sciences, Chifeng University, Chifeng. All experiments were performed according to the Regulations for the Administration of Affairs Concerning Experimental Animals.

### Animals, Experimental Design, and Diets

Forty-five 6-month-old Ujimqin lambs, one of the three most common varieties of coarse wool sheep in China, with a live BW of 29.50 ± 0.26 kg were used for the experiment. The lambs were weighed before morning feeding and randomly assigned to three diet treatments (three pens per treatment and five lambs per pen) with 30 square meters per pen in semi-open housing based on similar BW. Three diets were used in the experiment (Table 1). The lambs have received 30% alfalfa (HA, high alfalfa percentage group), 20% alfalfa (MA, moderate alfalfa percentage group), and 10% alfalfa (LA, low alfalfa percentage group), respectively (DM basis). The experiment consisted of a 15-day adaptation period and a 44-day data for sample collection. The feed was offered at 110% of their expected intake to ensure *ad libitum* feed intake. All lambs were fed twice daily at 800 and 1,600 h and were allowed free access to drinking water.

**TABLE 1:** Composition and nutrient contents of the experimental diet.

Item	HA	MA	LA
**Ingredients (%)**			
Alfalfa	30.00	20.00	10.00
Native grass	10.00	20.00	30.00
Maize	33.00	32.00	30.00
Wheat bran	12.00	11.00	11.00
Soybean meal	11.00	13.00	15.00
Salt	2.00	2.00	2.00
Mineral premix*^a^*	2.00	2.00	2.00
**Chemical compositions**			
Dry matter (%)	91.27	90.76	90.34
Organic matter (% DM)	96.93	96.87	96.82
Crude protein (% DM)	18.78	18.52	18.75
Ether extract (% DM)	1.70	1.09	1.77
Acid detergent fiber (% DM)	18.30	17.58	18.05
Neutral detergent fiber (% DM)	47.62	48.22	52.52

*^a^Composition of mineral premix. per kg, ferrum 170 g; cuprum 70 g; manganese 290 g; zinc 240 g; cobalt 510 mg; VA 1, 620, 000 IU; VD3 324, 000 IU; VE 540 IU; VK3 150 mg; VB1 60 mg; VB2 450 mg; VB5 1, 050 mg; VB12 0.9 mg. HA, high alfalfa percentages group; MA, middle alfalfa percentages group; LA, low alfalfa percentages group.*

### Data and Sample Collection

The amounts of feed offered and orts were recorded daily throughout the experiment to calculate voluntary feed intake and were expressed on a DM basis. All lambs were weighed in the morning (600–800 h) without fasting at the commencement and end of the experimental period and at 7-day intervals, and the BW gain was calculated as the difference between the final body weight and the initial body weight. At the end of the experiment, the lambs were transferred and slaughtered at a commercial slaughterhouse. All the lambs were slaughtered, and *Longissimus lumborum* muscle samples were collected from the carcass on the right side of the vertebrae for evaluation of meat quality, nutritional value, and fatty acid profile and stored in a freeze at –20°C until analysis. In addition, rumen samples were collected from all lambs, and the rumen content of each lamb in the same pen was first homogenized and then the same volume of rumen contents was mixed to reduce localized effects; a total of nine rumen samples were used for 16S rRNA sequencing. To obtain the rumen fluid samples, the whole rumen contents were strained through four layers of cheesecloth. Approximately, 30 ml of rumen fluid was collected in sterilized tubes. The rumen fluid samples were immediately frozen in liquid nitrogen and then stored at –80°C until further analysis.

### Feed Composition Analysis

Feed DM content was determined by drying samples for 48 h at 65°C. Then, the samples were ground through a 1-mm screen for the following analysis. The CP and ether extract (EE) contents were following the methods of the AOAC (2005). Neutral detergent fibers (NDF) and acid detergent fibers (ADF) were determined by the method of Van Soest et al. (1991) with an ANKOM A200i fiber analyzer (ANKOM Technology, Macedon, NY, United States) and were expressed exclusive of residual ash.

### Meat Quality Analysis

The moisture, protein, and intramuscular fat contents were determined according to the methods of the AOAC (2005). The pH_24_ of the longissimus lumborum muscle was measured between the 11th and 13th ribs using a glass electrode pH meter (STARTED 100/B, OHAUS, Shanghai, China). The water loss rate was determined as the percentage difference in weight following 24-h period during which samples were suspended within inflated plastic bags at 4°C (Forwood et al., 2021). The cooked meat rate was calculated according to the percentage difference in weight between pre- and post-cooked meat samples (Needham et al., 2019). The fatty acids methyl esters were measured according to the AOAC (2005) and Bu et al. (2021) methods with a gas chromatography–mass spectrometer 7890B (Agilent, California, United States).

### Bacterial DNA Extraction, Polymerase Chain Reaction Amplification, and 16S rDNA Sequencing

The DNA was extracted from samples using the E.Z.N.A.^®^ Stool DNA Kit (D4015, Omega, Inc., United States) according to the manufacturer’s instructions. Variable regions V3–V4 of the bacterial 16S rRNA gene were amplified with slightly modified versions of primers 341F (5′-CCTACGGGNGGCWGCAG-3′) and 805R (5′-GACTACHVGGGTATCTAATCC-3′) (Logue et al., 2016). The polymerase chain reaction (PCR) amplification and bioinformatics analysis were performed by LC-Bio Technology Co., Ltd. (Hangzhou, China). The 16S amplification was conducted according to the description of Zhou et al. (2022).

### Bioinformatics Analysis

Raw fastq files were subjected to quality control by UPARSE. Operational taxonomic units (OTUs) were clustered with 97% similarity by Usearch. The complexity of species diversity for samples using the Chao1, Shannon, and Simpson indexes and Good’s coverage analysis was also performed. Principal coordinate analysis (PCoA), Bray–Curtis similarity clustering, and abundance analysis were performed using R version 3.3.0 (Kong et al., 2021). The primary differentially abundant genera were analyzed by the linear discrimination analysis (LDA) coupled with the effect size (LEfSe) method (Segata et al., 2011). The PICRUSt was applied to predict metabolic genes based on the 16S rRNA data. Kyoto Encyclopedia of Genes and Genomes (KEGG) was also used to assign the genes into metabolic pathways. The heatmap package of R (R Core Team, 2014) was applied to generate heat maps of genera and Level 3-predicted microbial gene functions. Bar plots were generated in GraphPad Prism 7 (San Diego, CA, United States).

### Statistical Analysis

The growth performance, meat quality, and fatty acid profile were analyzed by one-way analysis of variance, and significant differences among treatments were declared at *P* < 0.05.

## Results

### Growth Performance

The growth performance and DMI are given in Table 2. Interestingly, the diet treatments had a significant (*P* < 0.05) influence on the DMI of lambs, and the DMI increased with the increasing percentages of native grass hay, while the significantly (*P* < 0.05) lower final BW and average daily gain were observed in the MA and LA groups compared with those in the HA group.

**TABLE 2:** Effects of diets with various levels of alfalfa and native grass hay on the growth performance of lambs.

Items	HA	MA	LA
Initial bodyweight (kg)	29.40 ± 0.37	29.60 ± 0.29	29.50 ± 0.69
Final bodyweight (kg)	40.60 ± 0.48*^a^*	38.60 ± 0.68*^b^*	38.60 ± 0.58*^b^*
Average daily gain (g)	25.45 ± 1.59*^a^*	20.45 ± 0.95*^b^*	20.68 ± 0.56*^b^*
Dry matter intake (kg)	1.38 ± 0.01*^c^*	1.49 ± 0.11*^b^*	1.54 ± 0.02*^a^*

*Within a row, values with different letters (a, b, and c) differ significantly (P < 0.05). HA, high alfalfa percentages group; MA, middle alfalfa percentages group; LA, low alfalfa percentages group.*

### Meat Quality

The meat quality of lambs fed on diets with various levels of alfalfa hay and native grass hay is given in Table 3. No significant (*P* > 0.05) difference was observed in pH_24_ in the three diets treatments. The water loss rate was significantly (*P* < 0.05) increased with the native grass percentages. Compared with the MA group, the cooked meat rate and moisture were significantly higher in the HA groups, while no significant (*P* > 0.05) difference was observed between the MA and LA groups. In addition, there was no significant (*P* > 0.05) difference in protein among the three diets treatment. Finally, the intramuscular fat (IMF) content in the LA group was significantly (*P* < 0.05) higher than that in the HA and MA groups, and there was no significant difference between the HA and MA groups.

**TABLE 3:** Effects of diets with various levels of alfalfa and native grass hay on the meat quality and nutritional value of lambs.

Item	HA	MA	LA
pH_24_	5.49 ± 0.16	5.57 ± 0.06	5.64 ± 0.03
Water loss rate (%)	20.90 ± 0.39*^c^*	24.03 ± 0.32*^b^*	26.72 ± 0.29*^a^*
Cooked meat rate (%)	59.21 ± 0.95*^a^*	55.39 ± 0.78*^b^*	56.77 ± 0.78*^ab^*
Moisture (%)	76.00 ± 0.20*^a^*	74.87 ± 0.26*^b^*	74.00 ± 0.44*^b^*
Protein (%)	22.83 ± 0.26	23.00 ± 0.45	23.67 ± 0.78
Intramuscular fat (%)	2.13 ± 0.04*^b^*	1.95 ± 0.12*^b^*	3.45 ± 0.29*^a^*

*Within a row, values with different letters (a, b, and c) differ significantly (P < 0.05). HA, high alfalfa percentages group; MA, middle alfalfa percentages group; LA, low alfalfa percentages group.*

Fatty acid compositions of *longissimus lumborum* muscle are shown in Table 4. As native grass hay percentages increased in the diet, the content of palmitic (C16:0) and palmitoleic (C16:1 *cis*-9) in the HA and MA groups were significantly (*P* < 0.05) lower than that in the LA groups, while no significant (*P* > 0.05) difference was observed between the HA and MA groups. In addition, compared with the HA group, the content of elaidic (C18:1 *trans*-9), oleic (C18:1 *cis*-9), and linoleic (C18:2 *cis*-9–*cis*-12) were significantly (*P* < 0.05) increased in the MA and LA groups. The content of α-linolenic (C18:3n3) was significantly (*P* < 0.05) higher in the LA group than that in the HA and MA groups.

**TABLE 4:** Fatty acid composition of *longissimus lumborum* muscle (mg/100 g fatty acid methyl esters).

Items	HA	MA	LA
Palmitic C16:0	522.02 ± 21.09*^b^*	573.83 ± 21.95*^b^*	691.21 ± 37.53*^a^*
Palmitoleic C16:1 *cis*-9	38.75 ± 1.66*^b^*	44.88 ± 2.98*^b^*	54.72 ± 2.86*^a^*
Stearic C18:0	334.69 ± 3.12*^b^*	368.35 ± 11.81*^ab^*	388.13 ± 15.66*^a^*
Elaidic C18:1 *trans*-9	37.83 ± 1.61*^c^*	45.98 ± 0.54*^b^*	90.87 ± 3.32*^a^*
Oleic C18:1 *cis*-9	721.58 ± 5.72*^c^*	900.68 ± 3.91*^b^*	966.85 ± 8.83*^a^*
Linolelaidic C18:2 *trans*-9–*trans*-12	6.32 ± 0.22	5.14 ± 0.73	5.21 ± 0.53
Linoleic C18:2 *cis*-9–*cis*-12	16.11 ± 36.67*^b^*	336.05 ± 4.50*^a^*	330.33 ± 12.61*^a^*
α-linolenic C18:3n3	19.84 ± 2.09*^b^*	19.11 ± 1.00*^b^*	28.28 ± 0.46*^a^*
Behenic C22:0	4.30 ± 0.20	4.81 ± 0.28	5.09 ± 0.28
Lignoceric C24:0	24.59 ± 2.44*^b^*	33.55 ± 0.92*^a^*	36.30 ± 0.84*^a^*

*Within a row, values with different letters (a, b, and c) differ significantly (P < 0.05). HA, high alfalfa percentages group; MA, middle alfalfa percentages group; LA, low alfalfa percentages group.*

### Rumen Microbiota

A total of 7,50,040 raw reads were obtained, with an average of 83,338 sequences for each rumen sample (data are not shown). As listed in Table 5, compared with the HA group, the alpha diversity results indicated that the MA and LA groups decreased the OTUs and Chao1 index. Good’s coverage index was higher than 99% in all samples, indicating the accuracy and reproducibility of the sequencing and adequate sequencing depth to investigate the dominant bacterial populations.

**TABLE 5:** Diversity indices of ruminal microbiota of lambs.

Items	HA	MA	LA
No. of operational taxonomic units (OTUs)	1,631.00 ± 28.04*^a^*	1,353.00 ± 35.68*^b^*	1,142.00 ± 42.29*^c^*
Chao1 index	1,632.22 ± 28.27*^a^*	1,456.01 ± 36.93*^b^*	1,142.21 ± 44.69*^c^*
Shannon index	8.51 ± 0.15*^a^*	8.43 ± 0.02*^a^*	7.88 ± 0.08*^b^*
Simpson index	0.99 ± 0.01	0.99 ± 0.01	0.98 ± 0.01
Good’s coverage index (%)	99.95 ± 0.03	99.94 ± 0.02	99.95 ± 0.02

*Within a row, values with different letters (a, b, and c) differ significantly (P < 0.05). HA, high alfalfa percentages group; MA, middle alfalfa percentages group; LA, low alfalfa percentages group.*

**FIGURE 1:**
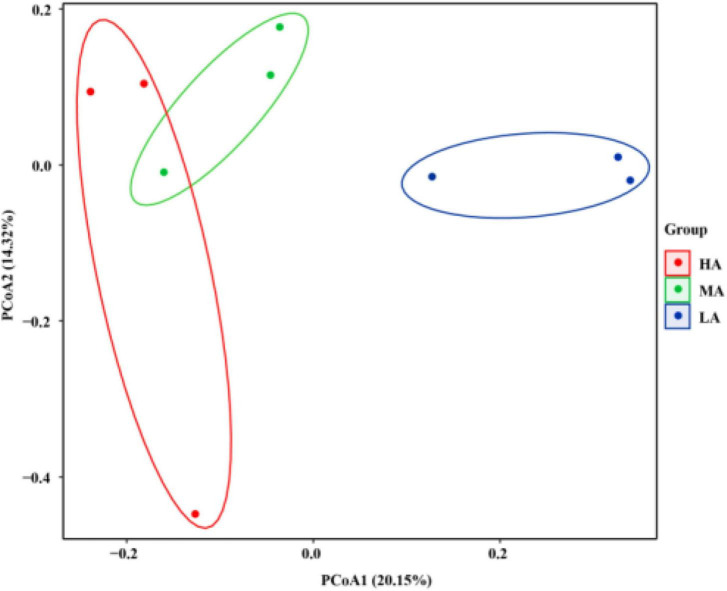
Principal coordinate analysis (PCoA) of rumen microbial communities based on unweighted UniFrac distance. HA, high alfalfa percentages group; MA, middle alfalfa percentages group; LA, low alfalfa percentages group.

In addition, the Venn diagram in the rumen samples showed that the groups shared 339 OTUs, while the HA, MA, and LA groups had 3,448; 2,778; and 2,157 exclusive OTUs, respectively (Supplementary Figure 1). To address the effects of alfalfa hay and native grass hay rations on beta diversity, unweighted UniFrac distance was used to characterize the bacterial community across all ruminal samples (Figure 1). The PCoA profile displayed that the composition of the bacterial community of these groups was distinctly separated from each other.

**FIGURE 2:**
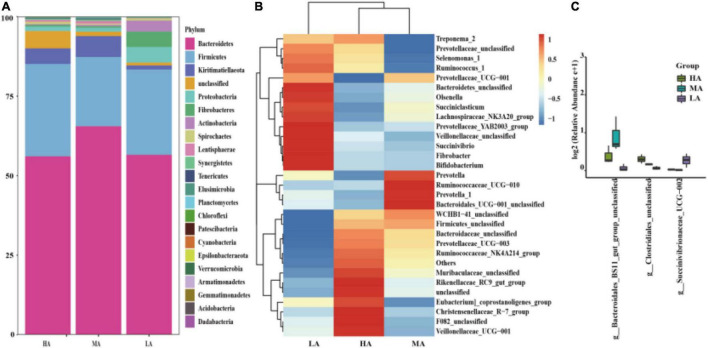
Classification of the bacterial community composition. **(A)** Phylum level. **(B)** Genus level. **(C)** Extended error bar plot showing the bacteria at the genus level that had significant differences among the HA, MA, and LA groups. HA, high alfalfa percentages group; MA, middle alfalfa percentages group; LA, low alfalfa percentages group.

Taxonomic analysis of the reads revealed the presence of 330 genera belonging to 23 phyla. At the phylum level, seven phyla were referred to as the detected phyla (relative abundance > 1% at least in one group). The most abundant phylum was *Bacteroidetes*, followed by *Firmicutes*, *Kiritimatiellaeota*, *Proteobacteria*, *Fibrobacteres*, *Actinobacteria*, and *Spirochaetes*, while no significant (*P* > 0.05) difference was observed among these groups (Figure 2A). At the genus level, 30 genera were considered as the detectable genera (relative abundance > 1% at least in one group). The main genera included *Prevotella_1*, *Muribaculaceae_unclassified*, *Rikenellaceae_RC9_gut_group*, *WCHB1-41_unclassified*, and *Bacteroidetes_unclassified* (Figure 2B). However, no significant (*P* < 0.05) difference was found among the dominant genera in the three groups, except *Bacteroidales_BS11_gut_group_unclassified*, *Clostridiales_unclassified*, and *Succinivibrionaceae_UCG-002* (Figure 2C).

**FIGURE 3:**
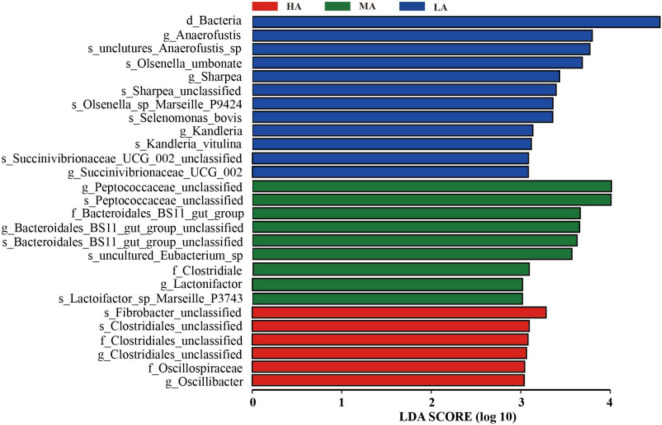
Linear discrimination analysis (LDA) coupled with effect size (LEfSe) analysis of the rumen microbial community of lamb in the HA, MA, and LA groups. Significantly different species with an LDA score greater than the estimated value (default score = 3). The length of the histogram represents the LDA score of different species in the three groups. HA, high alfalfa percentages group; MA, middle alfalfa percentages group; LA, low alfalfa percentages group.

As shown in Figure 3, LEfSe analysis revealed the difference in rumen microbiome among the three groups and the differences in the microbiome at various taxonomic levels with LDA scores. Specially, at the genus level, *Anaerofustis*, *Sharpea*, and *Kandleria* were enriched in the HA group, *Peptococcaceae_unclassified* and *Lactonifactor* were enriched in the MA group, while *Clostridiales_unclassified* and *Oscillibacter* were enriched in the LA group.

**FIGURE 4:**
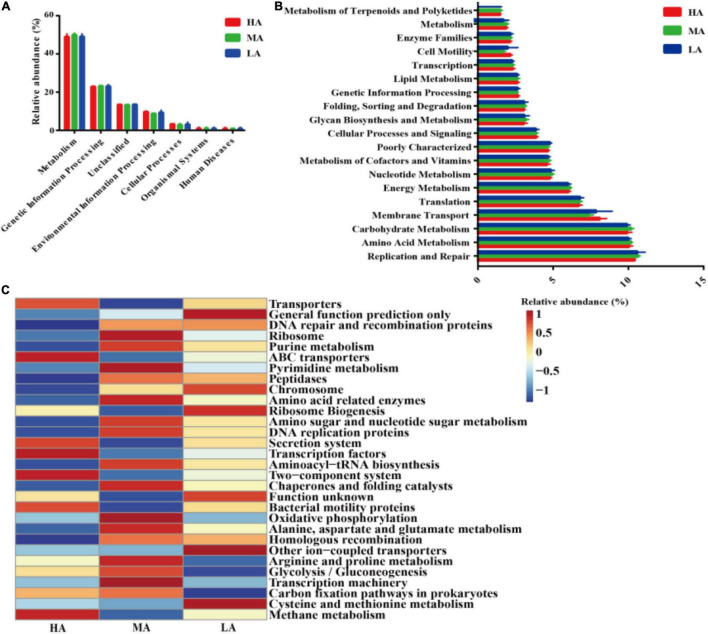
Dynamics of rumen bacterial functional profiles fed with different diets analyzed by PICRUSt (*n* = 3). **(A)** Level 1 metabolic pathways. **(B)** Level 2 Kyoto Encyclopedia of Genes and Genomes (KEGG) ortholog functional predictions of the relative abundances of the top 20 metabolic functions. **(C)** Level 3 KEGG ortholog functional predictions of the relative abundances of the top 30 metabolic functions. HA, high alfalfa percentages group; MA, middle alfalfa percentages group; LA, low alfalfa percentages group.

The microbial metabolic functions presented in Figure 4 were obtained based on the clusters of orthologous groups of the KEGG pathway database. A majority of the predicted protein sequences in the three diets among six different metabolic functions (Figure 4A) represented different pathways (Figure 4B). Notably, carbohydrate metabolism, replication and repair, and amino acid metabolism accounted for more than 10% of the enriched pathways among the three groups. Furthermore, carbohydrate metabolism, replication and repair, membrane transport, amino acid metabolism, translation, energy metabolism, poorly characterized nucleotide metabolism, and metabolism of cofactors and vitamins were significant in the HA group-fed lambs (*P* < 0.05). At level 3 of the microbial gene functions of bacteria, some differences in efficiency were observed (Figure 4C). The genus associated with carbon fixation pathways in prokaryotes was (*P* < 0.05) markedly enriched in the HA and MA groups, while the genus associated with cysteine and methionine metabolism was significantly enriched in the LA group.

### Correlation Between Rumen Microbiota and Growth Performance and Meat Quality

**FIGURE 5:**
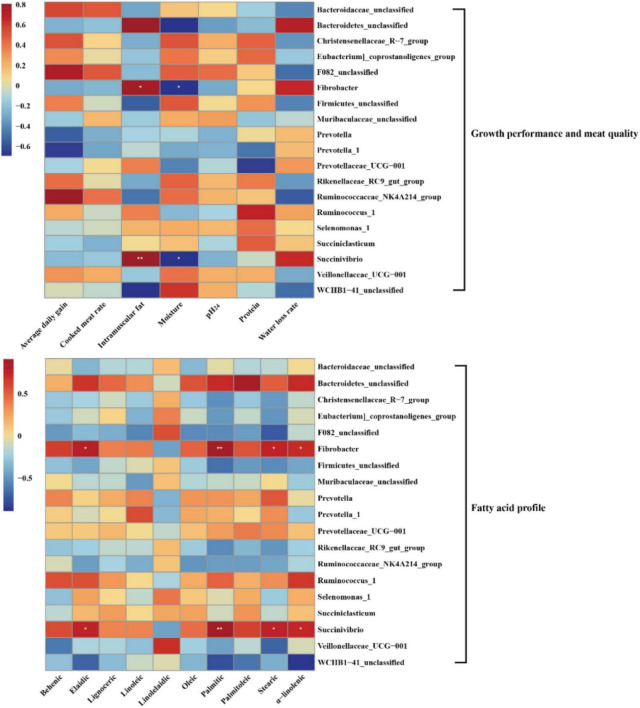
Heatmaps of Pearson’s correlations between dominant genera and growth performance, meat quality, and fatty acid profile. Red represents a positive correlation, while blue represents a negative correlation. Levels of significance are shown as follows: *P < 0.05; **P < 0.01.

Pearson’s correlation analysis was performed to further investigate the correlation between dominant microbial genera and growth performance, meat quality, and fatty acid profile (Figure 5). In the meat quality, the results of the present study showed that the genera *Fibrobacter* and *Succinivibrio* were positively associated with intramuscular fat, but negatively associated with moisture. In the fatty acid profile, the genera *Fibrobacter* and *Succinivibrio* were positively associated with palmitic (C16:0), stearic (C18:0), elaidic (C18:1 *trans*-9), and α-linolenic (C18:3n3). No significant difference was observed between the other genera and growth performance, meat quality, and fatty acid profile (*P* > 0.05).

## Discussion

This study characterized the changes in animal performance, meat quality, and rumen microbial community structure in response to replacing alfalfa with native grass at various ratios by multiple physicochemical analyses combined with 16S rRNA gene sequences, which was helpful in understanding the effects of replacing alfalfa with native grass on lambs.

The significantly increased voluntary intake with the increasing native grass in this study indicates that DM intake is determined mainly by the need for nutrients to meet energy requirements, following prior research that different starter intakes when fed various forage sources (Movahedi et al., 2016; Mirzaei et al., 2017), which could be explained by energy intake is the dominant factor that could influence DMI (Safari et al., 2009). In the present, final BW and growth rate decreased when ratios of native grass increased in the diet, which may be chiefly attributed to increased DMI throughout the experimental period (Mushi et al., 2009).

The parameter pH_24_ is widely used to evaluate raw meat quality, due to its strong association with meat quality characteristics, including color, water-holding capacity, and tenderness (Mordenti et al., 2019; Nunez-Sanchez et al., 2021). In this study, the pH_24_, ranging from 5.49 to 5.64, values were typically observed in sheep at the time of slaughter (Vergara and Gallego, 2000). Treatments had significant effects on the water loss rates and cooked meat rate, which could be explained by the final BW and pH values (Pearce et al., 2011).

Game meat is characterized as a low-fat and low-kilojoule product with an IMF content that is generally lower than 3% (Needham et al., 2020). The IMF content of meat is a key characteristic of meat quality due to its relation to the tenderness and flavor of meat, particularly during cooking or heat treatment (Purslow, 2005; Geldenhuys et al., 2014). The IMF content of meat in the LA group was significantly higher compared with that in the HA and MA groups, likely due to differences in dietary energy and intake (Needham et al., 2020). The meat is typically protein-rich and is one of the main nutrient sources for humans, besides the nutritional properties of meat that are largely attributed to its fat and FA contents (Marcon et al., 2020). The previous report indicated that balanced FA intakes are crucial to decrease the risk of atherosclerosis, cardiovascular, and other related diseases (Shahidi and Ambigaipalan, 2018). Therefore, the fatty acid composition of meat has been studied in depth because of its implications for human health (Calder, 2015; Francisco et al., 2020). In comparison with the lambs reared under three diets, the meat of LA lambs is characterized by higher major saturated fatty acids. This was connected with the increase of the major saturated fatty acid in meat, including palmitic acid (C16:0) and stearic acid (C18:0), which are significantly associated with coronary atherosclerotic heart disease risk and little cholesterol-raising effects in humans (Lanza et al., 2011). The different levels of saturated fatty acids, namely, palmitic and stearic, are likely caused by the significant difference in intake. The contents of elaidic (C18:1 *trans*-9) and oleic (C18:1 *cis*-9) increased with the increasing native grass in the diet. Prior research indicated that elaidic and oleic could reduce the risk of thrombosis and coronary heart disease coincidence, which is beneficial for human health (Marcon et al., 2020). In addition, a high concentration of α-Linolenic (C18:3n3) was also observed in the LA group. α-Linolenic (C18:3n3) is the precursor of long-chain n-3 fatty acids that have a wide range of biological effects as anti-inflammatory and cardioprotective effects (Marcon et al., 2020). These results could be a contribution to the complex compositions of native grass is benefit for deposition of n-6 and n-3 series (Aurousseaua et al., 2004). From the meat quality and fatty acid profile analysis, the meat from LA diet treatment is better than the others.

Despite diet as the known effect on growth performance, carcass traits, meat quality, and fatty acid profile were widely reported. Although rumen microbiota are relatively stable, they are highly responsive to changes in host genetics (Liu et al., 2020), age (Jami et al., 2013), feeding diet (Lin et al., 2021), and environmental factors (Uyeno et al., 2010). Of them, diet is the dominant factor in determining microbial community structure (Liu et al., 2020).

Our results suggest that replacing alfalfa with native grass could influence the rumen bacterial community composition in lambs. The higher OTUs number and Chao1 index were observed in the HA group compared with those in the MA and LA groups. The higher OTUs and Chao1 index observed in alfalfa than that in native grass may be the main reason (Bai et al., 2020; You et al., 2021). Significant differences were observed in the Shannon index among the three groups, suggesting that the HA group had higher community evenness than MA and LA groups.

The changes in the rumen bacterial compositions were also explored. Our results suggest that the HA, MA, and LA groups have their distinct microbiome, as reflected by the clustering of the samples by diet group using PCoA. Macroscopically, the different diets drove a separation in the bacterial community, the distinguishable changes among the three groups, following the reports that noticeable separation of the microbial structure was observed among forage, grain, and concentrate diets (Trabi et al., 2019; Lin et al., 2021), which could be a contribution to the growth of microorganisms under various pH conditions (Hobson and Purdom, 1959; Li et al., 2017).

The phylum-level core microbiomes were *Bacteroidetes*, *Firmicutes*, *Proteobacteria*, and *Kiritimatiellaeota*, with accounting for approximately 90% of bacterial species (Anderson et al., 2017; Guo et al., 2020; Chai et al., 2021). In addition, the genus-level dominant bacteria were *Prevotella 1*, *Muribaculaceae_unclassified*, *Rikenellaceae_RC9_gut_group*, etc., which are following the findings of previous studies (Li et al., 2020b; Zhou et al., 2022); these genera were not affected by the diet in this study. *Bacteroidetes* and *Firmicutes* were the two most abundant and active bacterial community members involved in the degradation of carbohydrates and proteins (Xue et al., 2020). The primary role of *Bacteroidetes* is connected to degrading diverse plant polysaccharides and improving the nutrient utilization of the host to enhance the host’s immunity (Bayliss and Houston, 1984; Bäckhed et al., 2005). *Firmicutes* is another important role in the degradation of fiber and cellulose and is associated with the decomposition of polysaccharide and the utilization of energy (Crisol-Martínez et al., 2017; Liang et al., 2021). In this study, the bacterial richness and taxonomic composition in the HA, MA, and LA groups were similar, and no significant difference was observed at the phylum level. The stability of the most abundant bacteria may reflect the presence of the core microbiome (Henderson et al., 2015). In this study, no significant difference was observed in the primary genera among the three treatments. Meanwhile, some taxa had different relative abundance at the genus level, a finding consistent with other reports (Scharen et al., 2017; Li et al., 2020a), and likely reflecting specialized niches related to the digestion of dietary fiber (Shen et al., 2020).

*Bacteroidales_BS11_gut_group* are specialized to break down hemicellulose monomeric sugars by fermentation and produce short-chain fatty acids that are vital for ruminant energy (Solden et al., 2017; Liu et al., 2019). Meanwhile, the higher abundance of *Bacteroidales_BS11_gut_group* was higher in the MA group compared with that in the HA and LA groups. The intermediate disturbance and specialized niches may be the main reason (Liang et al., 2020; Shen et al., 2020). *Clostridium* contains common free-living bacteria, as well as important pathogens (Sun et al., 2016). The consequent decreased abundance of *Clostridium* may benefit the health of the intestine of lambs in the MA and LA groups, which could be a contribution to the complex compositions of native grass. Prior research also indicated that rumen bacterial compositions are directly linked to the diet; these included changes in the *Succinivibrionaceae* family that changed in relative abundance in diet-related changes (Furman et al., 2020). Therefore, a significant difference was found in the genus *Succinivibrionaceae_UCG-002*. In addition, *Succinivibrionaceae* is considered a key determinant in methane emissions (Wallace et al., 2019). Replacing alfalfa with native grass may be friendly to the environment and their effects need to be researched in further study.

Various bacteria were correlated with growth performance, meat quality, and fatty acid profile, revealing that multiple physicochemical parameters were active by rumen microbiota. In this study, the significantly higher abundance of *Fibrobacter* and *Succinivibrio* and higher IMF were observed in the LA group, which could be explained by the higher abundance of *Fibrobacter* in grass-fed lambs, high efficiency in degrading crystalline cellulose, and a high ability to solubilize plant cell wall polysaccharides (Fernando et al., 2010; Xie et al., 2018). Furthermore, the genus *Succinivibrio* is positively correlated with fat percentage (Hailemariam et al., 2020). It is well known that cellulolytic bacteria were a benefit for fatty acid synthesizing, including *Ruminococcus*, *Fibrobacter*, and *Succinivibri*o (Zhang et al., 2020). Therefore, the genera *Fibrobacter* and *Succinivibrio* were also positively associated with palmitic (C16:0), stearic (C18:0), elaidic (C18:1 *trans*-9), and α-linolenic (C18:3n3). These results indirectly explain an observation of the correlation between rumen microbiota and growth performance, meat quality, and fatty acid profile.

## Conclusion

This study shows how multiple physicochemical analyses combined with 16S rRNA gene sequences may be used in observing changes and influences of replacing alfalfa with native grass. The results showed that replacing alfalfa with native grass could directly affect animal performance, meat quality, and fatty acid profile. The meat quality and fatty acid profile analysis revealed that replacing alfalfa hay with native grass hay is more beneficial for Mongolian lambs, and the meat from LA diet treatment is better than the others. In addition, correlation analysis of the association of rumen microbiome with growth performance, meat quality, and fatty acid profile provides us with a comprehensive understanding of the composition and function of rumen microbiota. One of the limitations of this study was the lack of rumen fermentation parameters (such as pH, VFA, and ammonia). Regardless, the findings of this study provide the knowledge of how the diet affects animal performance, meat quality of lambs, and microbiota of the rumen and lay a theoretical basis for replacing alfalfa with native grass.

## Data Availability Statement

The datasets presented in this study can be found in online repositories. The names of the repository/repositories and accession number(s) can be found below: NCBI–PRJNA8000534.

## Ethics Statement

The animal study was reviewed and approved by the College of Chemistry and Life Sciences, Chifeng University.

## Author Contributions

SD involved in investigation, methodology, visualization, validation, data curation, writing—original draft, and conceptualization. SY involved in investigation, software, formal analysis, and writing—review and editing. LS involved in formal analysis. XW involved in data curation. YJ and YZ involved in conceptualization, funding acquisition, project administration, and supervision. All authors contributed to the article and approved the submitted version.

## Conflict of Interest

The authors declare that the research was conducted in the absence of any commercial or financial relationships that could be construed as a potential conflict of interest.

## Publisher’s Note

All claims expressed in this article are solely those of the authors and do not necessarily represent those of their affiliated organizations, or those of the publisher, the editors and the reviewers. Any product that may be evaluated in this article, or claim that may be made by its manufacturer, is not guaranteed or endorsed by the publisher.

## References

[B1] Anderson C. L. Sullivan M. B. Fernando S. C. (2017). Dietary energy drives the dynamic response of bovine rumen viral communities. *Microbiome* 5 155. 10.1186/s40168-017-0374-3 29179741 PMC5704599

[B2] AOAC (2005). *Official Methods of Analysis*, 18th Edn. Gaithersburg, MD: AOAC International.

[B3] Aurousseaua B. Bauchart D. Calichon E. Micol D. Priolo A. (2004). Effect of grass or concentrate feeding systems and rate of growth on triglyceride and phospholipid and their fatty acids in the M. longissimus thoracis of lambs. *Meat Sci.* 66 531–541. 10.1016/S0309-1740(03)00156-6 22060862

[B4] Bäckhed F. Ley R. E. Sonnenburg J. L. Peterson D. A. Gordon J. I. (2005). Host-bacterial mutualism in the human intestine. *Science* 307 1915–1920. 10.1126/science.1104816 15790844

[B5] Bai J. Xu D. Xie D. Wang M. Li Z. Guo X. (2020). Effects of antibacterial peptide-producing *Bacillus subtilis* and *Lactobacillus buchneri* on fermentation, aerobic stability, and microbial community of alfalfa silage. *Bioresour. Technol.* 315:123881. 10.1016/j.biortech.2020.123881 32731157

[B6] Bayliss C. E. Houston A. P. (1984). Characterization of plant polysaccharide- and mucin-fermenting anaerobic bacteria from human feces. *Appl. Environ. Microbiol.* 48 626–632. 10.1128/AEM.48.3.626-632.1984 6093693 PMC241577

[B7] Bickhart D. M. Weimer P. J. (2018). Symposium review: host-rumen microbe interactions may be leveraged to improve the productivity of dairy cows. *J. Dairy Sci.* 101 7680–7689. 10.3168/jds.2017-13328 29102146

[B8] Bu Z. K. Ge G. T. Jia Y. S. Du S. (2021). Effect of hay with or without concentrate or pellets on growth performance and meat quality of Ujimqin lambs on the inner Mongolian Plateau. *Anim. Sci. J.* 92:e13553. 10.1111/asj.13553 33938599

[B9] Calder P. C. (2015). Functional roles of fatty acids and their effects on human health. *JPEN J. Parenter. Enteral Nutr.* 39 18–32. 10.1177/0148607115595980 26177664

[B10] Chai J. Lv X. Diao Q. Usdrowski H. Zhuang Y. Huang W. (2021). Solid diet manipulates rumen epithelial microbiota and its interactions with host transcriptomic in young ruminants. *Environ. Microbiol.* 23 6557–6568. 10.1111/1462-2920.15757 34490978 PMC9292864

[B11] Crisol-Martínez E. Stanley D. Geier M. S. Hughes R. J. Moore R. J. (2017). Understanding the mechanisms of zinc bacitracin and avilamycin on animal production: linking gut microbiota and growth performance in chickens. *Appl. Microbiol. Biot.* 101 4547–4559. 10.1007/s00253-017-8193-9 28243710

[B12] Du S. You S. H. Bao J. Ge G. T. Jia Y. S. Cai Y. M. (2020). Growth performance, carcass characteristics, and meat quality of Mongolian lambs fed native grass or hay with or without concentrate on the inner Mongolian Plateau. *Can. J. Anim. Sci.* 100 470–478. 10.1139/cjas-2019-0126

[B13] Fernando S. C. Purvis H. T. Najar F. Z. Sukharnikov L. O. Krehbiel C. R. Nagaraja T. G. (2010). Rumen microbial population dynamics during adaptation to a high-grain diet. *Appl. Environ. Microbiol.* 76 7482–7490. 10.1128/AEM.00388-10 20851965 PMC2976194

[B14] Forwood D. L. Holman B. W. B. Hopkins D. L. Smyth H. E. Hoffman L. C. Chaves A. V. (2021). Feeding unsaleable carrots to lambs increased performance and carcass characteristics while maintaining meat quality. *Meat Sci.* 173:108402. 10.1016/j.meatsci.2020.108402 33316707

[B15] Francisco A. E. Janicek M. Dentinho T. Portugal A. P. V. Almeida J. M. Alves S. P. (2020). Effects of alfalfa particle size and starch content in diets on feeding behaviour, intake, rumen parameters, animal performance and meat quality of growing lambs. *Meat Sci.* 161:107964. 10.1016/j.meatsci.2019.107964 31683223

[B16] Furman O. Shenhav L. Sasson G. Kokou F. Honig H. Jacoby S. (2020). Stochasticity constrained by deterministic effects of diet and age drive rumen microbiome assembly dynamics. *Nat. Commun.* 11:1904. 10.1038/s41467-020-15652-8 32312972 PMC7170844

[B17] Geldenhuys G. Homan L. C. Muller M. (2014). Sensory profiling of Egyptian goose (*Alopochen aegyptiaca*) meat. *Food Res. Int.* 64 25–33. 10.1016/j.foodres.2014.06.005 30011648

[B18] Guo W. Zhou M. Ma T. Bi S. Wang W. Zhang Y. (2020). Survey of rumen microbiota of domestic grazing yak during different growth stages revealed novel maturation patterns of four key microbial groups and their dynamic interactions. *Anim. Microbiome* 2:23. 10.1186/s42523-020-00042-8 33499950 PMC7807461

[B19] Hailemariam S. Zhao S. G. Wang J. Q. (2020). Complete genome sequencing and transcriptome analysis of nitrogen metabolism of Succinivibrio dextrinosolvens strain Z6 isolated from dairy cow rumen. *Front. Microbiol.* 11:1826. 10.3389/fmicb.2020.01826 33013723 PMC7507024

[B20] Hao X. Y. Gao H. Wang X. Y. Zhang G. N. Zhang Y. G. (2017). Replacing alfalfa hay with dry corn gluten feed and Chinese wild rye grass: effects on rumen fermentation, rumen microbial protein synthesis, and lactation performance in lactating dairy cows. *J. Dairy Sci.* 100 2672–2681. 10.3168/jds.2016-11645 28215882

[B21] Henderson G. Cox F. Ganesh S. Jonker A. Young W. Global R. C. C. (2015). Rumen microbial community composition varies with diet and host, but a core microbiome is found across a wide geographical range. *Sci. Rep.* 5, 14567–14581. 10.1038/srep14567 26449758 PMC4598811

[B22] Hobson P. N. Purdom M. R. (1959). Gram-negative sporing bacterium from the rumen. *Nature* 138 904–905. 10.1038/183904a0 13644245

[B23] Jami E. Israel A. Kotser A. Mizrahi I. (2013). Exploring the bovine rumen bacterial community from birth to adulthood. *ISME J.* 7 1069–1079. 10.1038/ismej.2013.2 23426008 PMC3660679

[B24] Jin D. Y. Murray P. J. Xin X. P. Qin Y. F. Chen B. R. Qing G. L. (2018). Attribution of explanatory factors for change in soil organic carbon density in the native grasslands of inner Mongolia. *China. J. Arid Land.* 10 375–387. 10.1007/s40333-018-0056-4

[B25] Kong C. Yan X. Liu Y. Haung L. Zhu Y. He J. (2021). Ketogenic diet alleviates colitis by reduction of colonic group 3 innate lymphoid cells through altering gut microbiome. *Sig. Transduct. Target. Ther.* 6 154. 10.1038/s41392-021-00549-9 33888680 PMC8062677

[B26] Lanza M. Fabro C. Scerra M. Bella M. Pagano R. Brogna D. M. R. (2011). Lamb meat quality and intramuscular fatty acid composition as affected by concentrates including different legume seeds. *Ital. J. Anim. Sci.* 10 87–94. 10.4081/ijas.2011.e18

[B27] Li F. Wang Z. Dong C. Li F. Wang W. Yuan Z. (2017). Rumen bacteria communities and performances of fattening lambs with a lower or greater subacute ruminal acidosis risk. *Front. Microbiol.* 8:2506. 10.3389/fmicb.2017.02506 29312208 PMC5733016

[B28] Li Y. Zhang G. N. Feng G. Z. Lv J. Y. Fang X. P. Zhao C. (2020a). Effects of replacing alfalfa hay with malt sprouts and corn stover on milk production and nitrogen partitioning in dairy cows. *Anim. Feed Sci. Tech.* 270:114701. 10.1016/j.anifeedsci.2020.114701

[B29] Li Z. Mu C. Xu Y. Shen J. Zhu W. (2020b). Changes in the solid-, liquid-, and epithelium-associated bacterial communities in the rumen of Hu lambs in response to dietary urea supplementation. *Front. Microbiol.* 11:244. 10.3389/fmicb.2020.00244 32153533 PMC7046558

[B30] Li Y. Zhang G. N. Xu H. J. Zhou S. Dou X. J. Lin C. (2019). Effects of replacing alfalfa hay with *Moringa oleifera* leaves and peduncles on intake, digestibility, and rumen fermentation in dairy cows. *Livest. Sci.* 220 211–216. 10.1016/j.livsci.2019.01.005

[B31] Liang J. S. Zheng W. G. Zhang H. B. Zhang P. Y. Cai Y. J. Wang Q. Y. (2021). Transformation of bacterial community in rumen liquid anaerobic digestion of rice straw. *Environ. Pollut.* 269:116130. 10.1016/j.envpol.2020.116130 33261966

[B32] Liang Y. T. Xiao X. Nuccio Y. Y. Yuan M. T. Zhang N. Xue K. (2020). Differentiation strategies of soil rare and abundant microbial taxa in response to changing climatic regimes. *Environ. Microbiol.* 22 1327–1340. 10.1111/1462-2920.14945 32067386

[B33] Lin L. Trabi E. B. Xie F. Mao S. (2021). Comparison of the fermentation and bacterial community in the colon of Hu sheep fed a low-grain, non-pelleted, or pelleted high-grain diet. *Appl. Microbiol. Biotechnol.* 105 2071–2080. 10.1007/s00253-021-11158-5 33559720

[B34] Liu C. Wu H. Liu S. Chai S. Meng Q. Zhou Z. (2019). Dynamic alterations in Yak rumen bacteria community and metabolome characteristics in response to feed type. *Front. Microbiol.* 10:1116. 10.3389/fmicb.2019.01116 31191470 PMC6538947

[B35] Liu H. Hu L. Han X. Zhao N. Xu T. Ma L. (2020). Tibetan sheep adapt to plant phenology in alpine meadows by changing rumen microbial community Structure and function. *Front. Microbiol.* 11:587558. 10.3389/fmicb.2020.587558 33193243 PMC7649133

[B36] Logue J. B. Stedmon C. A. Kellerman A. M. Nielsen N. J. Andersson A. F. Laudon H. (2016). Experimental insights into the importance of aquatic bacterial community composition to the degradation of dissolved organic matter. *ISME. J.* 10 533–545. 10.1038/ismej.2015.131 26296065 PMC4817675

[B37] Marcon H. Baldissera M. D. Furlan V. J. M. Wagner R. Alba D. F. Molosse V. L. (2020). Curcumin supplementation positively modulates fatty acid profiles in lamb meat. *Small Ruminant Res.* 190 106141. 10.1016/j.smallrumres.2020.106141

[B38] Mirzaei M. Khorvash M. Ghorbani G. R. Kazemi-Bonchenari M. Ghaffari M. H. (2017). Growth performance, feeding behavior, and selected blood metabolites of Holstein dairy calves fed restricted amounts of milk: no interactions between sources of finely ground grain and forage provision. *J. Dairy Sci.* 100 1086–1094. 10.3168/jds.2016-11592 28012617

[B39] Mordenti A. L. Brogna N. Canestrari G. Bonfante E. Eusebi S. Mammi L. M. E. (2019). Effects of breed and different lipid dietary supplements on beef quality. *Anim. Sci. J.* 90 619–627. 10.1111/asj.13177 30821084

[B40] Movahedi B. Foroozandeh A. D. Shakeri P. (2016). Effects of different forage sources as a free-choice provision on the performance, nutrient digestibility, selected blood metabolites and structural growth of Holstein dairy calves. *J. Anim. Physiol. Anim. Nutr.* 101 293–301. 10.1111/jpn.12527 27277573

[B41] Mushi D. E. Safari J. Mtenga L. A. Kifaro G. C. Eik L. O. (2009). Effects of concentrate levels on fattening performance, carcass and meat quality attributes of Small East African × Norwegian crossbred goats fed low quality grass hay. *Livest. Sci.* 124 148–155. 10.1016/j.livsci.2009.01.012

[B42] Needham T. Engels R. A. Bures D. Kotrba R. van Rensburg B. J. Hoffman L. C. (2020). Carcass yields and physiochemical meat quality of semi-extensive and intensively farmed impala (*Aepyceros melampus*). *Foods* 9:418. 10.3390/foods9040418 32260057 PMC7230698

[B43] Needham T. Laubser J. G. Kotrba R. Bures D. Smyth H. Hoffman L. C. (2019). Sensory characteristics of the longissimus thoracis et lumborum and biceps femoris muscles from male and female common eland (*Taurotragus oryx*). *Meat Sci.* 158:107918. 10.1016/j.meatsci.2019.107918 31450093

[B44] Nunez-Sanchez N. Ramirez C. A. Blanco F. P. Gomez-Cortes P. de la Fuente M. A. Amor M. V. (2021). Effects of algae meal supplementation in feedlot lambs with competent reticular groove reflex on growth performance, carcass traits and meat characteristics. *Foods* 10:857. 10.3390/foods10040857 33920806 PMC8071124

[B45] Pearce K. L. Rosenvold K. Andersen H. J. Hopkins D. L. (2011). Water distribution and mobility in meat during the conversion of muscle to meat and ageing and the impacts on fresh meat quality attributes-a review. *Meat Sci.* 89 111–124. 10.1016/j.meatsci.2011.04.007 21592675

[B46] Purslow P. P. (2005). Intramuscular connective tissue and its role in meat quality. *Meat Sci.* 70 435–447. 10.1016/j.meatsci.2004.06.028 22063743

[B47] R Core Team (2014). *R: A Language and Environment for Statistical Computing*. Vienna: R Foundation for Statistical Computing.

[B48] Rosenberg E. Zilber-Rosenberg I. (2018). The hologenome concept of evolution after 10 years. *Microbiome* 6:78. 10.1186/s40168-018-0457-9 29695294 PMC5922317

[B49] Safari J. Mushi D. E. Mtenga L. A. Kifaro G. C. Eik L. O. (2009). Effects of concentrate supplementation on carcass and meat quality attributes of feedlot finished small East African goats. *Livest. Sci.* 125 266–274. 10.1016/j.livsci.2009.05.007

[B50] Scharen M. Kiri K. Riede S. Gardener M. Meyer U. Hummel J. (2017). Alterations in the rumen liquid-, particle- and epithelium-associated microbiota of dairy cows during the transition from a silage- and concentrate-based ration to pasture in spring. *Front. Microbiol.* 8:744. 10.3389/fmicb.2017.00744 28512453 PMC5411454

[B51] Segata N. Izard J. Waldron L. Gevers D. Miropolsky L. Garrett W. (2011). Metagenomic biomarker discovery and explanation. *Genome Boil.* 12:R60. 10.1186/gb-2011-12-6-r60 21702898 PMC3218848

[B52] Shahidi F. Ambigaipalan P. (2018). Omega-3 polyunsaturated fatty acids and their health benefits. *Annu. Rev. Food Sci. Technol.* 9 345–381. 10.1146/annurev-food-111317-095850 29350557

[B53] Shen J. S. Li Z. P. Yu Z. T. Zhu W. Y. (2020). Effects of dietary replacement of soybean meal with dried distillers grains with solubles on the microbiota occupying different ecological niches in the rumen of growing Hu lambs. *J. Anim. Sci. Biotechno.* 11:93. 10.1186/s40104-020-00499-2 32939263 PMC7487462

[B54] Solden L. M. Hoyt D. W. Collins W. B. Plank J. E. Daly R. A. Hildebrand E. (2017). New roles in hemicellulosic sugar fermentation for the uncultivated bacteroidetes family BS11. *ISME J.* 11 691–703. 10.1038/ismej.2016.150 27959345 PMC5322302

[B55] Sun Y. Su Y. Zhu W. (2016). Microbiome-metabolome responses in the cecum and colon of pig to a high resistant starch diet. *Front. Microbiol.* 7:779. 10.3389/fmicb.2016.00779 27303373 PMC4880592

[B56] Trabi E. B. Seddik H. E. Xie F. Lin L. M. Mao S. Y. (2019). Comparison of the rumen bacterial community, rumen fermentation and growth performance of fattening lambs fed low grain, pelleted or non-pelleted high grain total mixed ration. *Anim. Feed. Sci. Technol.* 253 1–12. 10.1016/j.anifeedsci.2019.05.001

[B57] Uyeno Y. Sekiguchi Y. Tajima K. Takenaka A. Kurihara M. Kamagata Y. (2010). An rRNA-based analysis for evaluating the effect of heat stress on the rumen microbial composition of Holstein heifers. *Anaerobe* 16 27–33. 10.1016/j.anaerobe.2009.04.006 19446029

[B58] Van Soest J. P. Robertson J. B. Lewis B. A. (1991). Methods for dietary fiber, neutral detergent fiber and non-starch polysaccharides in relation to animal nutrition. *J. Dairy Sci.* 74 3583–3597. 10.3168/jds.S0022-0302(91)78551-2 1660498

[B59] Vergara H. Gallego L. (2000). Effect of electrical stunning on meat quality of lamb. *Meat Sci.* 56 345–349. 10.1016/S0309-1740(00)00061-9 22062164

[B60] Wallace R. J. Sasson G. Garnsworthy P. C. Tapio I. Gregson E. Bani P. (2019). A heritable subset of the core rumen microbiome dictates dairy cow productivity and emissions. *Sci. Adv.* 5:eaav8391. 10.1126/sciadv.aav8391 31281883 PMC6609165

[B61] Xie X. Yang C. Guan L. Wang J. Xue M. Liu J. (2018). Persistence of cellulolytic bacteria fibrobacter and *Treponema* after short-term corn stover-based dietary intervention reveals the potential to improve rumen fibrolytic function. *Front. Microbiol.* 9:1363. 10.3389/fmicb.2018.01363 29997589 PMC6029512

[B62] Xue M. Y. Sun H. Z. Wu X. H. Liu J. X. Guan L. L. (2020). Multi-omics reveals that the rumen microbiome and its metabolome together with the host metabolome contribute to individualized dairy cow performance. *Microbiome* 8:64. 10.1186/s40168-020-00819-8 32398126 PMC7218573

[B63] You S. H. Du S. Ge G. T. Wan T. Jia Y. S. (2021). Microbial community and fermentation characteristics of native grass prepared without or with isolated lactic acid bacteria on the Mongolian Plateau. *Front. Microbiol.* 12:731770. 10.3389/fmicb.2021.731770 34659159 PMC8517267

[B64] Zhang Z. A. Niu X. L. Li F. Li F. D. Guo L. (2020). Ruminal cellulolytic bacteria abundance leads to the variation in fatty acids in the rumen digesta and meat of fattening lambs. *J. Anim. Sci.* 98 1–8. 10.1093/jas/skaa228 32687154 PMC7448102

[B65] Zhou Y. L. Sun L. Cheng Q. M. Li Y. C. Chen J. X. Zhao B. (2022). Effect of pelleted alfalfa or native grass total mixed ration on the rumen bacterial community and growth performance of lambs on the Mongolian Plateau. *Small Ruminant Res.* 207:106610. 10.1016/j.smallrumres.2021.106610

