# Supplementary material for: Effects of Replacing Alfalfa Hay With Native Grass Hay in Pelleted Total Mixed Ration on Physicochemical Parameters, Fatty Acid Profile, and Rumen Microbiota in Lamb
Source: Front Microbiol. 2022 Apr 29;13:861025. doi: 10.3389/fmicb.2022.861025 (PMC9106545; doi:10.3389/fmicb.2022.861025)
Supplement: Supplementary file 1 [file Image_1.pdf]

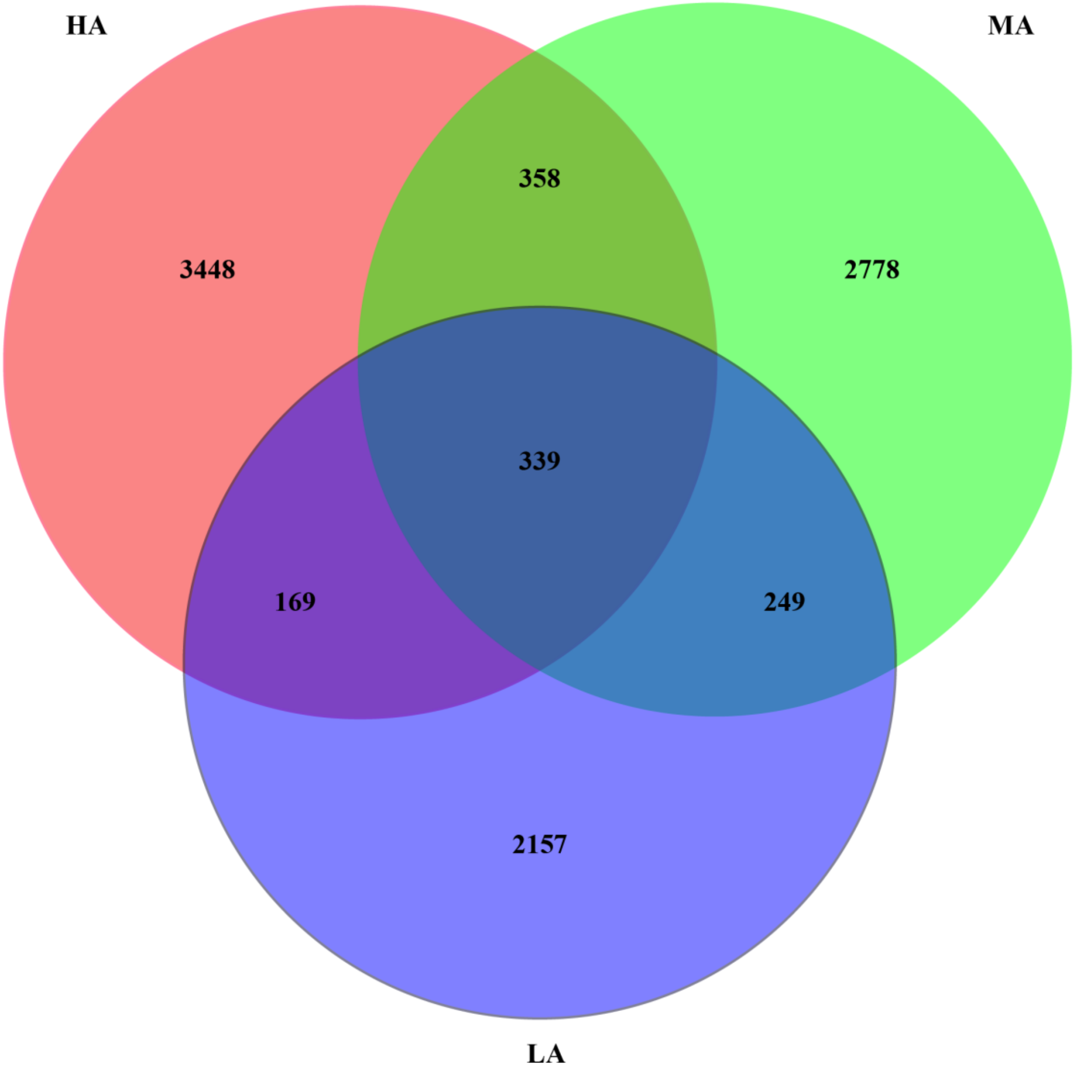

Supplementary Fig. 1. Venn diagram representing the common and unique OTUs found at each diet. HA, high alfalfa percentages group; MA, middle alfalfa percentages group; LA, low alfalfa percentages group.
